# β-sitosterol ameliorates myocardial infarction injury via modulating the NF-κB and necroptosis signaling pathways

**DOI:** 10.3389/fphar.2025.1719074

**Published:** 2026-01-06

**Authors:** Jia-jia Xu, Min-wei Chen, Long-zhen Lai, Di Xiao, Shu-yan Jiang, Jun-xin Lin, Zheng-hao Zhang, Zhong-gui Shan

**Affiliations:** 1 Department of Cardiovascular Surgery, The First Affiliated Hospital of Xiamen University, School of Medicine, Xiamen University, Xiamen, China; 2 Department of Hematology, People’s Hospital of Xinjiang Uygur Autonomous Region, Urumqi, China

**Keywords:** cardioprotection, myocardial infarction, necroptosis, NF-κB signaling, β-sitosterol

## Abstract

**Introduction:**

Acute myocardial infarction (MI) is a leading global cause of morbidity and mortality, where inflammatory response and programmed cell death (PCD) are critical in disease progression. β-sitosterol (β-SITO), a phytosterol with known cardioprotective effects, has been implicated in cardiovascular diseases, but its specific role and mechanisms in MI remain underexplored.

**Methods:**

This study employed both *in vivo* and *in vitro* models. Male C57BL/6J mice with MI were used to evaluate the effects of β-SITO treatment. Cardiac function was assessed via echocardiography, infarct size and fibrosis were analyzed histologically. *In vitro*, cardiomyocyte viability under hypoxia and TGF-β-induced cardiac fibroblast activation were examined. Mechanistic insights were gained through transcriptomic profiling, molecular docking studies, and validation by Western blotting.

**Results:**

β-SITO treatment significantly reduced myocardial infarct size, alleviated cardiac fibrosis, and improved cardiac function in MI mice. *In vitro*, it enhanced cardiomyocyte viability under hypoxia and inhibited TGF-β-induced fibroblast activation. Transcriptomic analysis revealed that β-SITO modulated pathways related to immune-inflammatory responses, NF-κB, and necroptosis signaling. Molecular docking confirmed its strong binding affinity to key components of these pathways. Western blotting validated the inhibition of NF-κB activation and necroptosis in both hypoxic cardiomyocytes and MI mouse heart tissue.

**Conclusion:**

β-SITO demonstrates significant therapeutic potential for improving post-MI recovery. Its cardioprotective effects are likely mediated through the modulation of NF-κB and necroptosis signaling pathways, highlighting it as a promising candidate for MI treatment.

## 1 Introduction

As a prevalent cardiovascular disorder, acute myocardial infarction (MI) remains a leading global health burden, accounting for significant morbidity and mortality worldwide (Zhao et al., 2019; Roth et al., 2020). In general, MI triggers a pathological cascade characterized by coronary artery occlusion, leading to acute myocardial ischemia and hypoxia (Chapman et al., 2025). Dysregulated immune modulation is a key driver of post-MI inflammation. During the acute phase of MI, pro-inflammatory immune cells infiltrate the infarcted myocardium, initiating a robust inflammatory cascade (Ma et al., 2025). Prolonged inflammation not only impairs cardiac repair but also exacerbates cardiomyocyte apoptosis, necrosis, and fibrotic remodeling, ultimately contributing to adverse ventricular remodeling and progressive cardiac dysfunction (Wang et al., 2025a). Programmed cell death (PCD) is a hallmark pathological feature of MI, significantly influencing disease progression (Galluzzi et al., 2018). Given its critical role, therapeutic strategies targeting anti-inflammatory pathways and inhibition of cardiomyocyte death are essential to mitigate post-MI injury (Yang et al., 2022a; Wang et al., 2025b).

Emerging evidence highlights the central involvement of RIPK3-dependent necroptosis and NF-κB signaling in post-MI pathophysiology (Chai et al., 2023; Zhang et al., 2025a; Maslov et al., 2022). Necroptosis, a regulated form of necrosis mediated by RIPK1/RIPK3 and MLKL, is increasingly recognized as a major contributor to ischemic injury (Luedde et al., 2014). Necroptosis not only induces membrane rupture and cell lysis but also facilitates the release of danger-associated molecular patterns (DAMPs), thereby creating a feed-forward loop that intensifies inflammatory responses following MI (Zhang et al., 2019). Previous studies have reported substantial activation of RIPK3 and MLKL in ischemic myocardium, implicating necroptosis as a therapeutic target for attenuating myocardial injury and adverse left ventricular remodeling (Wu et al., 2025a). The NF-κB signaling pathway is another central regulator of MI pathophysiology (Shan et al., 2024). As a master transcriptional controller of inflammatory mediators, NF-κB activation occurs rapidly after ischemic insult and contributes to the production of cytokines, chemokines, and adhesion molecules that propagate inflammatory cell recruitment (Zhuang et al., 2023; Zhang et al., 2025b). Importantly, recent evidence indicates extensive crosstalk between NF-κB signaling and necroptosis. NF-κB can modulate the expression of RIP kinases, while RIPK1/RIPK3 activation may further stimulate NF-κB-dependent inflammatory cascades, jointly aggravating myocardial injury (Zhang et al., 2023; Alghibiwi et al., 2025). Despite these insights, the integrated roles of NF-κB and RIPK3-dependent necroptosis in MI remain incompletely understood, and therapeutic approaches targeting both pathways simultaneously are still lacking.

Pharmacological modulation of these pathways may offer promising therapeutic avenues to attenuate excessive inflammation and improve cardiac outcomes. To date, reperfusion therapy remains the cornerstone of MI management, with coronary recanalization demonstrating significant prognostic benefits (Wei et al., 2025). However, the concomitant ischemia/reperfusion (I/R) injury paradoxically exacerbates myocardial damage, resulting in impaired cardiac function and increased risk of severe complications (Yan et al., 2025). While contemporary therapeutic strategies have improved acute outcomes, they remain insufficient to prevent subsequent ventricular remodeling and heart failure progression (Zhu et al., 2025). This critical limitation underscores the urgent need to develop novel treatment modalities that can fundamentally reverse post-AMI heart failure.

Accumulating clinical studies have validated the historical application of Traditional Chinese Medicine in cardiovascular therapeutics, demonstrating measurable treatment benefits (Lu et al., 2019; Sun et al., 2024). β-sitosterol (abreviated as β-SITO in this study), a prominent member of the phytosterol family, is widely distributed in numerous medicinal plant species, including *Aconiti Lateralis Radix Praeparata* (Yang et al., 2022b), *Trema orientalis* (Mekarunothai et al., 2024), bitter melon (Kim et al., 2025), *Angelica sinensis* (Chen et al., 2025a), *Curcuma longa L.* (Wang et al., 2025c), *S. surattense* (Yang et al., 2025), and soybean (Yang et al., 2024). Previous studies have reported that β-SITO has various biological actions, including anti-inflammation (Wu et al., 2025b), hepatoprotection (Zhang et al., 2024; Wang et al., 2025d), neuroprotection (Dolrahman and Thong-Asa, 2024), cardioprotective properties (Jaiswal et al., 2024; Adhimoolam et al., 2024), anti-diabetes (Liu et al., 2024), anti-oxidation (Gupta et al., 2011), and anti-tumor effects (Wang et al., 2024a; Chen et al., 2024; Wang et al., 2025e). A study by Wang *et al* proven that β-SITO triggers ovarian cancer apoptosis via ASS1-mediated Nrf2 degradation and PTEN/PI3K/AKT-dependent ROS generation (Wang et al., 2024b). Additionally, β-SITO demonstrated significant anti-diabetic effects in rat models of type 2 diabetes induced by high-fat diet and sucrose. Their study revealed that β-SITO can exert reno-protective effects by maintaining cellular homeostasis, balancing apoptosis, and reinforcing antioxidant activity. Mechanistically, β-SITO targets the TGF-β1/Nrf2/SIRT1/p53 pathways, suggesting its therapeutic utility in diabetic nephropathy and related metabolic disorders (Jayaraman et al., 2025). It is worth noting that some studies have confirmed that β-SITO can effectively prevent and treat cardiovascular diseases. Jiang and colleagues indicated that β-SITO attenuates atherosclerosis by upregulating catalase, which suppresses the PI3K/Akt/mTOR pathway, thereby reducing lipid accumulation and VSMC phenotypic switching (Jiang et al., 2024). Moreover, β-SITO exhibits cardioprotective effects in a monocrotaline-induced rat model of pulmonary hypertension. Studies indicated that β-SITO ameliorates pulmonary arterial hypertension by modulating vascular smooth muscle cell phenotype switching and suppressing DNA damage-mediated cGAS/STING signaling (Li et al., 2024). Nevertheless, the precise effects of β-SITO on myocardial infarction pathogenesis and recovery remain poorly characterized. In this research, through integrated *in vitro* and *in vivo* approaches, we explored β-SITO’s therapeutic potential against myocardial infarction and deciphered its mechanistic basis.

## 2 Materials and methods

### 2.1 Materials

β-sitosterol (C_29_H_50_O) with over 96% purity was obtained from Sigma-Aldrich (S9889, soluble in ethanol, sonication-assisted dissolution, United States). Hematoxylin Eosin (H&E) stain kit (G1005), Masson’s Trichrome stain kit (G1006), wheat germ agglutinin (WGA) staining (GDP1020) and TTC staining (G1017) were purchased from Service-bio (Wuhan, China).

### 2.2 Construction of MI model and treatment protocol

Male C57BL/6J mice of 8–12 weeks were used in the experiments. The mice were randomly allocated to four groups: Sham + Vehicle, MI + Vehicle, MI + β-SITO (10 mg/kg), MI + β-SITO (50 mg/kg). After acclimation, the MI models were established in mouses. Briefly, Mice were anesthetized with isoflurane, and the surgical site was aseptically prepared by depilating the cervical region extending to the mid-sternal area using electric clippers. A thoracotomy was performed through the 3rd or 4th intercostal space using ophthalmic curved forceps under a stereomicroscope. The left anterior descending coronary artery (LAD) was identified and ligated approximately 1.5 mm distal to the inferior edge of the left auricle. After ligation, residual air within the thoracic cavity was evacuated, followed by layered closure of the incision. Sham-operated mice underwent the same procedure, including LAD exposure and threading, but without ligation. After that, the mice were orally fed with 200 mL β-SITO for 28 consecutive days. Following euthanasia, tissue specimens were harvested for subsequent analysis.

### 2.3 Echocardiography determination

Briefly, cardiac function was evaluated using the VEVO2100 high-resolution ultrasound system (Visual-Sonics, Canada). Under continuous 1%–2% isoflurane anesthesia, murine chest hair was removed with depilatory cream. M-mode echocardiography was performed to obtain left ventricular functional parameters.

### 2.4 Histopathological analysis

Cardiac tissues were fixed, paraffin-embedded, and sectioned (5 μm) for histological analysis. Myocardial architecture, fibrosis, and hypertrophy were evaluated by H&E, Masson’s trichrome, and WGA staining, respectively. Digital whole-slide images were acquired using a Leica Aperio Versa 200 scanning system (Germany), with cardiomyocyte cross-sectional area (WGA) and collagen deposition (Masson) quantified using ImageJ software.

### 2.5 TTC staining

Myocardial infarct size was measured by TTC staining. In short, the mice were anesthesia with 5% isoflurane and then sacrificed, hearts were excised and sectioned transversely into 1 mm slices using a precision matrix. For infarct quantification, slices were incubated in 1% TTC (Solarbio, China) for 10 min, with the reaction terminated by 4% paraformaldehyde. The myocardial infarct area was imaged under a stereomicroscope (Zeiss Stemi 508, Germany) and calculated by ImageJ.

### 2.6 Cell isolation and culture

Primary neonatal mouse cardiomyocytes (NMCMs) and cardiac fibroblasts (NMCFs) were obtained as previously described (Yang et al., 2023). In brief, NMCMs and NMCFs were isolated from 1 to 3 days-old C57BL/6 mice. Following surface disinfection with 75% ethanol, hearts were aseptically excised and transferred to a laminar flow hood. Cardiac tissues were enzymatically dissociated using 0.25% trypsin (Service-bio) and type II collagenase (Thermo-Fisher Scientific) in sequential digestions.

Cell suspensions were centrifuged (1,500 × g, 5 min) and resuspended in complete high-glucose DMEM (Thermo-Fisher Scientific) supplemented with 10% FBS (Thermo-Fisher Scientific) and 1% penicillin/streptomycin. NMCMs and NMCFs were separated by differential adhesion (1–2 h at 37 °C). The adherent population (NMCFs) and non-adherent supernatant (NMCMs) were maintained in complete medium under standard culture conditions (37 °C, 5% CO_2_). For experimental treatments, cells were exposed to: 10 ng/mL TGF-β (Sigma-Aldrich), β-SITO (10 μM or 40 μM), Hypoxic conditions (5% CO_2_, 95% N_2_ at 37 °C).

### 2.7 Cell viability assay

Cell viability was assessed using the Cell Counting Kit-8 (CCK-8) assay. Briefly, cells were seeded in 96-well plates (1 × 10^4^ cells/well) and cultured for 24 h prior to treatment with hypoxia (5% CO_2_/95% N_2_), TGF-β (10 ng/mL), and/or β-SITO at serial concentrations for 24 h. Following treatment, 10 μL CCK-8 reagent was added per well, incubated for 3 h, and absorbance was measured at 450 nm using a microplate reader.

### 2.8 Lactate dehydrogenase (LDH) release assay

NMCMs were plated in 96-well plates and treated with TGF-β (10 ng/mL) and/or β-SITO (10 or 40 μM) as indicated. Following treatment, culture supernatants were collected and analyzed for LDH release using a commercial cytotoxicity detection kit (C0017, Beyotime Institute of Biotechnology) according to the manufacturer’s instructions.

### 2.9 Transwell migration assay

Cell migration was assessed using Transwell chambers (8 μm pore size; Millipore, Billerica, MA). Transfected cells (2 × 10^4^) in serum-free medium were seeded in the upper chamber, while the lower chamber contained DMEM supplemented with 10% FBS as a chemoattractant. Following 24 h incubation, migrated cells on the membrane underside were fixed with 4% paraformaldehyde, stained with 0.1% crystal violet, and quantified by counting ten random fields at ×200 magnification using an inverted light microscope (Olympus IX73).

### 2.10 Immunofluorescence (IF)

For immunofluorescence analysis, NMCFs grown on coverslips were treated with β-SITO/TGF-β for 24 h. After fixation (4% paraformaldehyde, 30 min) and permeabilization (0.1% Triton X-100, 15 min), nonspecific sites were blocked with 2% BSA. Primary antibody incubation (anti-α-SMA, ab7817, abcam) was performed overnight at 4 °C, followed by fluorescent secondary antibody (1 h, RT). Nuclear staining with DAPI (C0065, Solarbio) preceded imaging on an Olympus IX73 system.

### 2.11 Transcriptomics analysis

The transcriptomics analysis was performed by Hangzhou Guangke Ande Biotechnology Co., LTD. In short, total RNA was extracted from NMCMs using standard extraction methods. Then, RNA quality assessment was performed using a Nanodrop ND-2000 spectrophotometer and Agilent 2,100 Bioanalyzer. Sequencing libraries were prepared using the ABclonal mRNA-seq Library Preparation Kit following manufacturer’s specifications, with library quantification and size distribution verification conducted on an Agilent 4,150 TapeStation system. High-throughput sequencing was performed on an Illumina Nova-seq 6,000 platform (Applied Protein Technology), generating 150 bp paired-end reads. Raw data processing and differential expression analysis were executed using DESeq2, with significantly differentially expressed genes (DEGs) defined as those exhibiting >2-fold change at adjusted p-value <0.05. Lastly, the enrichment analyses of differential genes were implemented following the procedures as previously described by Gao et al. (2025).

### 2.12 Molecular docking

The structures of NLRP3, RIPK3, and MLKL were obtained from the PDB database with respective ID 7LFH, 6OKO, and 4BTF. The models for P65 and SRC3 were generated using AlphaFold3 (Abramson et al., 2024). And then prepared structurally using the Protein Preparation Wizard module of Schrödinger (Release 2021–2) (Napolitano and Ballabio, 2016). All operating parameters were set using the software default settings unless otherwise specified. The protein structure preparation process includes removing water molecules from the native structure, adding hydrogen atoms, removing crystalline solvent molecules, completing missing residues and loops using Prime (Jacobson et al., 2002; Jacobson et al., 2004), optimizing the hydrogen bond networks, and energy minimization of the structure using the OPLS4 force field (Lu et al., 2021). The compound β-Sitosterol used for docking was prepared using the LigPrep (LigPrep, Schrödinger, LLC, New York, NY, 2021) tool with default parameters. Maestro (Maestro, Schrödinger, LLC, New York, NY, 2021) and PyMol (The PyMOL Molecular Graphics System, Version 2.3 Schrödinger, LLC) were used for structural visualization and docking result profiling.

### 2.13 Quantitative reverse Transcription-PCR

In brief, total RNA isolation from samples was performed with TRIzol reagent (Invitrogen), followed by cDNA synthesis using a reverse transcription system (Transcriptor First Strand cDNA Synthesis Kit, Thermo-Fisher). Quantitative PCR analysis was conducted with SYBR Green Master Mix (Thermo-Fisher) on a Step-One-Plus Real-Time PCR System (Applied Biosystems). Gene-specific primer sequences are detailed in Supplementary Table S1.

### 2.14 Protein extraction and Western blotting

Protein extraction and immunoblotting were conducted as follows: Cardiac tissues and NMCMs were homogenized in RIPA lysis buffer supplemented with protease and phosphatase inhibitors. Protein quantification was performed using a BCA assay. Equal protein aliquots were electrophoresed on 8%–12% gradient SDS-polyacrylamide gels and subsequently transferred to PVDF membranes. After blocking with 5% non-fat dried milk in TBST for 1 h at room temperature, membranes were probed with specific primary antibodies targeting: GAPDH (60004-1-Ig, Protein-tech), NF-κB p65 (8242S, CST), phosphorylated p65 (3033S, CST), NLRP3 (ab214185, Abcam), RIPK3 (ab56164, Abcam), phospho-RIPK3 (ab195117, Abcam), MLKL (ab184718, Abcam), phospho-MLKL (ab196436, Abcam), and IL-1β (12242S, CST). Following overnight incubation at 4 °C, membranes were exposed to horseradish peroxidase (HRP)-conjugated secondary antibodies for 1 h at room temperature. Protein-antibody complexes were visualized using the ECL detection system and quantified by densitometry.

### 2.15 Statistical analysis

All quantitative data are presented as mean ± standard deviation (SD). Statistical comparisons were conducted using one-way analysis of variance (ANOVA) followed by Tukey’s post-hoc test for multiple comparisons (GraphPad Prism 8.0.1; GraphPad Software, San Diego, CA). A two-tailed *p*-value < 0.05 was considered statistically significant.

## 3 Results

### 3.1 β-SITO improves myocardial injury and cardiac function in MI mouses

**FIGURE 1:**
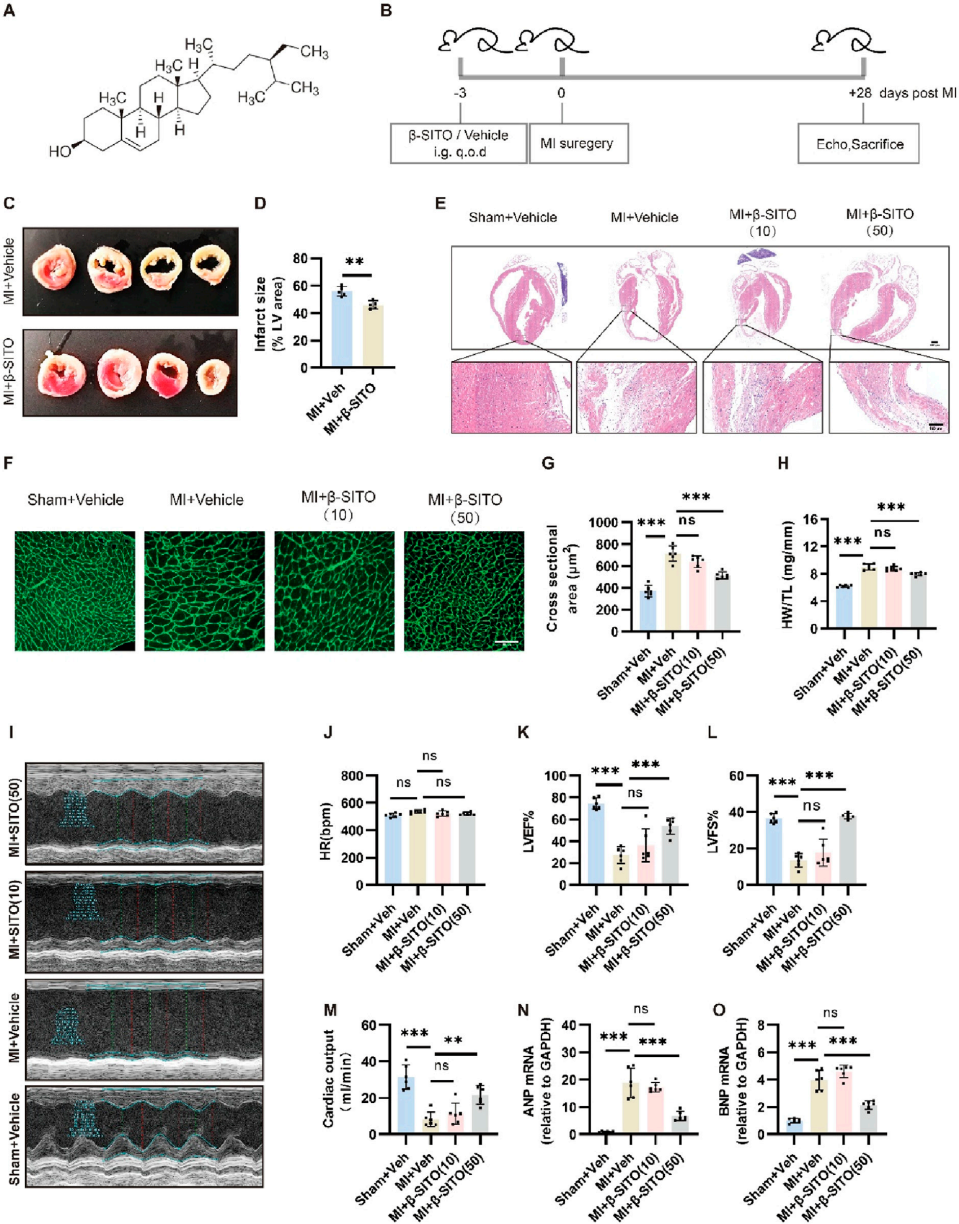
β-SITO improves MI-induced myocardial injury and cardiac function *in vivo*. **(A)** The chemical structural formula of β-SITO. **(B)** Schematic representation of the murine MI experimental protocol. **(C,D)** Myocardial infarct size was measured by TTC staining in MI + Vehicle and MI + β-SITO (50 mg/kg) group. Quantitation of myocardial infarct area were calculated by ImageJ (n = 5). **(E)** Representative images of HE staining of the myocardial tissues. Scale bar = 1,000 or 100 μm. **(F,G)** Representative WGA-stained left ventricular sections across experimental groups. Scale bar = 100 μm. Quantitative analysis of cardiomyocyte cross-sectional area (n = 6). **(H)** Ratio of heart weight (HW) to tibia length (TL) in different groups (n = 6). **(I)** Representative echocardiographic images across experimental groups. **(J–M)** Quantitative analysis of cardiac function parameters, including heart rate (HR), left ventricular ejection fraction (LVEF), fractional shortening (LVFS), Cardiac output (n = 6). **(N,O)** The myocardial mRNA expression of ANP and BNP was quantified by real-time quantitative PCR (RT-qPCR), with GAPDH serving as the endogenous control (n = 6). **P < 0.01, ***P < 0.001; ns means no significance.

The molecular structure of β-SITO is presented in Figure 1A. To investigate its cardioprotective potential, we established a myocardial infarction (MI) model in male C57BL/6J mice through permanent coronary artery ligation. Postoperative animals received daily β-SITO treatment (10 or 50 mg/kg, oral gavage) for 28 days (Figure 1B), with physiological and histological endpoints evaluated. At first, the TTC staining was performed on the tissue slices to measure myocardial infarct size (Figures 1C,D), and the results showed that β-SITO effectively decreased infarct areas in MI mice compared to the Vehicle group. Furthermore, H&E and WGA staining revealed that MI mice developed significant cellular edema and cardiomyocyte hypertrophy, which were attenuated by β-SITO treatment (Figures 1E–G). Besides, HW/TL ratios were significantly reduced in the β-SITO treated group compared to MI controls (Figure 1H). Meanwhile, echocardiography was used to determine cardiac function in various mice groups. As described in Figures 1I–M, our results manifested that a significant decrease in LVEF, LVFS and cardiac output after MI induction, when compared with those in the sham group. By contrast, treatment with β-SITO remarkably enhanced left ventricular function. Consistent with these above results, RT-qPCR results further proven that the mRNA expression of heart failure-related proteins (ANP and BNP) were obviously reduced in the β-SITO treated group when compared with the MI group (Figures 1N,O). From all above results, we revealed that β-SITO effectively improved myocardial injury and cardiac function *in vivo*.

### 3.2 β-SITO ameliorates cardiac fibrosis in MI mouses

**FIGURE 2:**
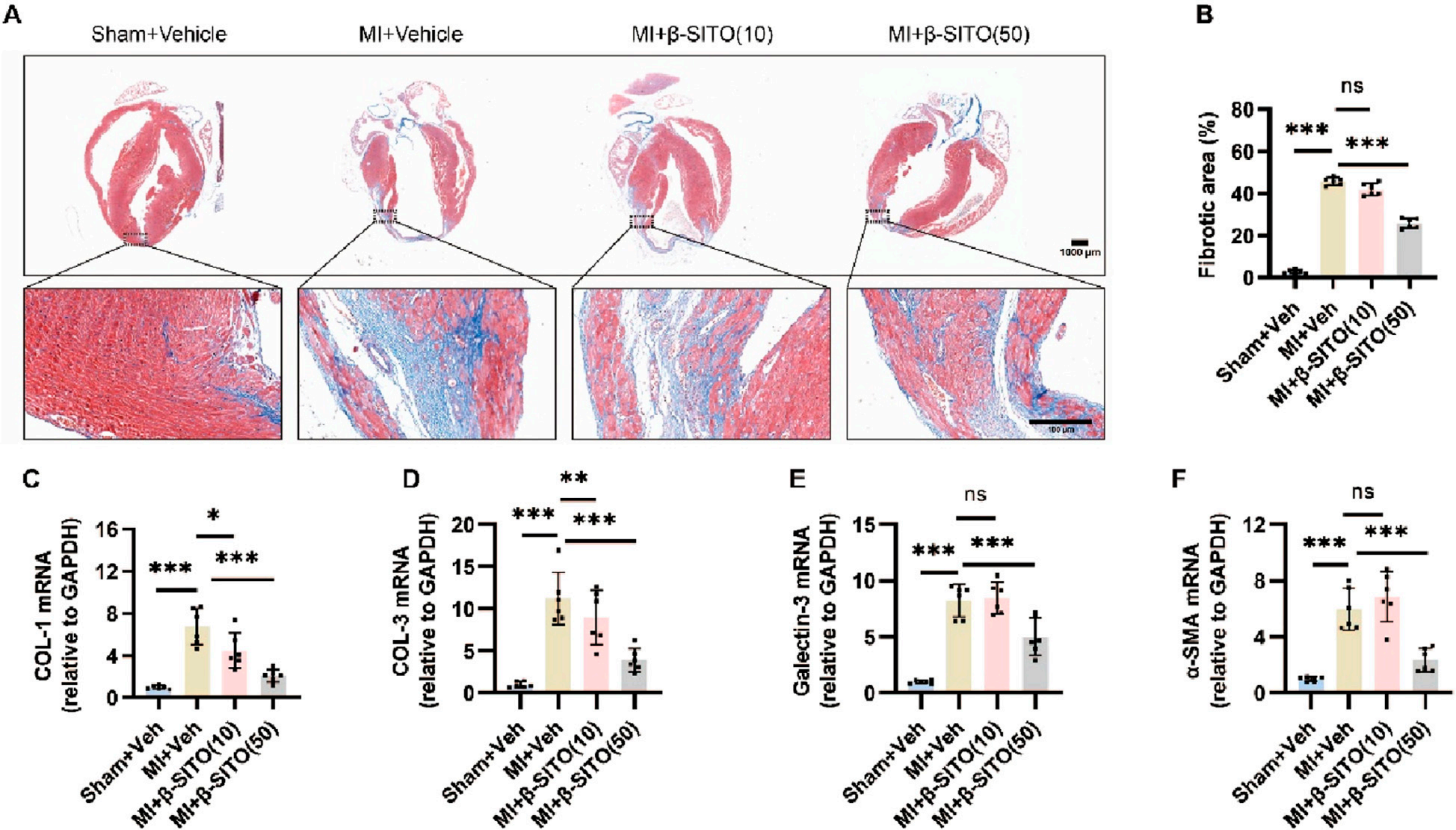
β-SITO ameliorates cardiac fibrosis in MI mouses. **(A,B)** Masson trichrome staining (left) and quantification of the scar area (right) at 28 days after MI (n = 6, scale bar = 1,000 or 100 μm). **(C–F)** The myocardial mRNA expression of COL-1, COL-3, Galectin-3 and α-SMA was quantified by RT-qPCR, with GAPDH serving as the endogenous control (n = 6). *P < 0.05, **P < 0.01, ***P < 0.001; ns means no significance.

To assess the effect of β-SITO on MI-triggered cardiac fibrosis, collagen deposition was quantified using Masson staining. As shown in Figures 2A,B, β-SITO treatment considerably reduced cardiac fibrosis in the left ventricle compared to the MI group. Additionally, the mRNA expression of COL-1, COL-3, Galectin-3 and α-SMA in MI mouses was higher than that in the sham group. This trend was reversed to varying degrees following β-SITO treatment (Figures 2C–F). Collectively, these findings indicated that β-SITO can inhibit cardiac fibrosis in MI mouses.

### 3.3 β-SITO alleviates HO-induced cardiomyocytes damage *in vitro*

**FIGURE 3:**
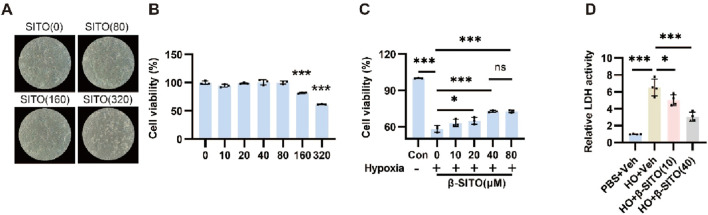
β-SITO alleviates HO-induced cardiomyocytes damage *in vitro*. **(A)** Representative images of NMCMs after 24 h β-SITO exposure (0, 80, 160, and 320 μM). **(B)** NMCM viability following β-SITO treatment (0, 10, 20, 40, 80, 160, and 320 μM, 24 h) was quantified via CCK-8 assay (n = 3). **(C)** NMCMs were exposed to β-SITO (0, 10, 20, 40, and 80 μM, 24 h) under normoxic or hypoxic conditions, with cell viability assessed using CCK-8 assay (n = 3). **(D)** LDH release was measured in NMCMs following 24 h β-SITO (0, 10, and 40 μM) treatment under normoxic or hypoxic conditions (n = 4). *P < 0.05, **P < 0.01, ***P < 0.001; ns means no significance.

Next, NMCMs were used to evaluate β-SITO’s effects on cardiomyocyte injury. As depicted in Figures 3A,B, CCK-8 assay demonstrated that Low-concentration β-SITO exhibited negligible cytotoxicity in NMCMs. Concurrently, β-SITO treatment significantly enhanced NMCM viability under hypoxic conditions (Figure 3C). Moreover, as illustrated in Figure 3D, the experimental results demonstrated that β-SITO dose-dependently inhibited LDH release in NMCMs. Altogether, these above results certified that β-SITO significantly alleviated HO-induced cardiomyocytes damage *in vitro.*

### 3.4 β-SITO ameliorates the activation of NMCFs induced by TGF-β *in vitro*

**FIGURE 4:**
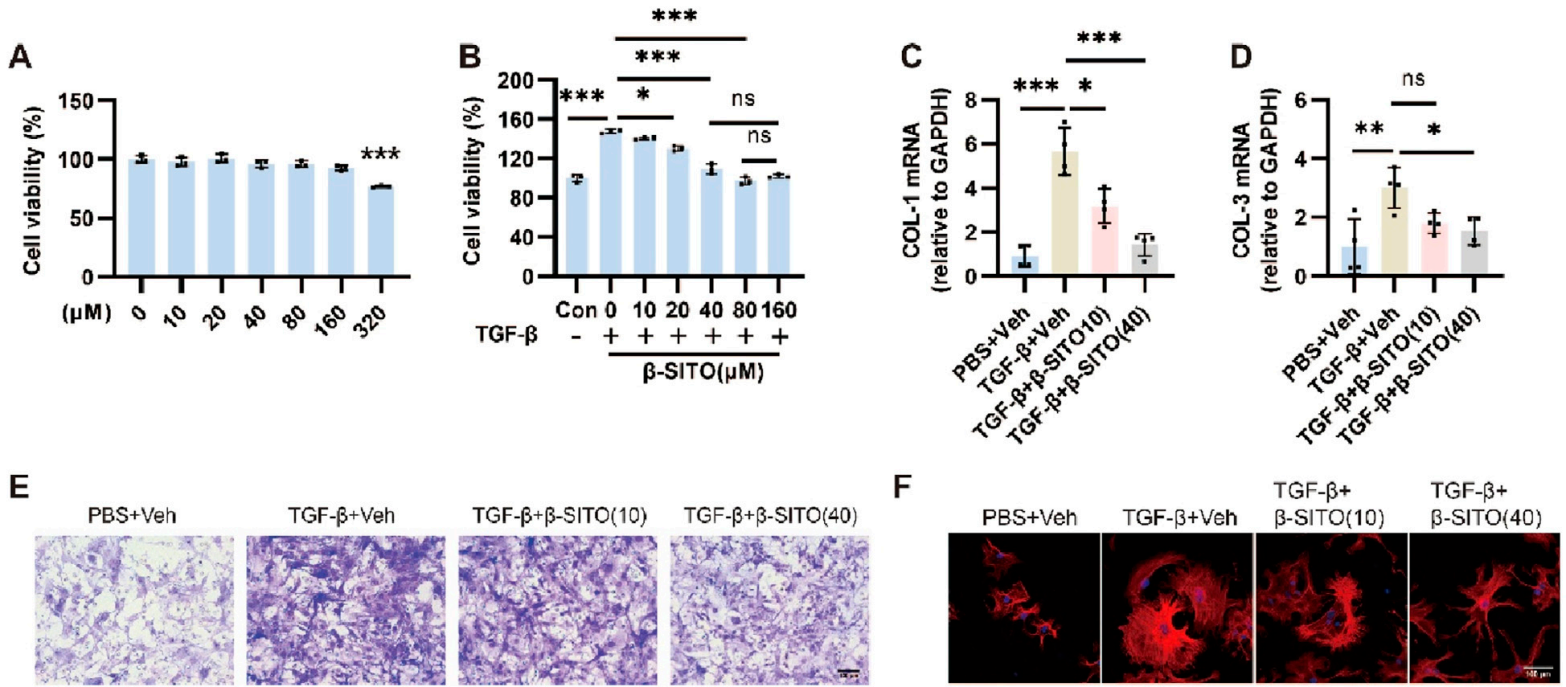
β-SITO alleviates the activation of NMCFs induced by TGF-β *in vitro.* **(A)** NMCFs viability following β-SITO treatment (0, 10, 20, 40, 80, 160, and 320 μM, 24 h) was quantified via CCK-8 assay (n = 3). **(B)** NMCFs were exposed to β-SITO (0, 10, 20, 40, 80 and 160 μM, 24 h) in the presence of TGF-β (10 ng/mL) or saline, with cell viability assessed using CCK-8 assay (n = 3). **(C,D)** The mRNA expression of COL-1 and COL-3 was quantified by RT-qPCR in NMCFs with or without β-SITO treatment, with GAPDH serving as the endogenous control (n = 4). **(E)** Representative map of cell migration (scale bar = 100 μm). **(F)** NMCFs were exposed to β-SITO (10 and 40 μM, 24 h) in the presence of TGF-β (10 ng/mL) or saline. DAPI (blue) and α-SMA (red) staining were performed in NMCFs. Immunofluorescence photographs were obtained using an inverted fluorescence microscope (scale bar = 100 μm). *P < 0.05, **P < 0.01, ***P < 0.001; ns means no significance.

TGF-β serves as a pivotal regulator of fibroblast-to-myofibroblast transition, driving enhanced proliferative capacity, collagen deposition, and migratory activity. For that reason, we next estimated the effects of β-SITO on the migratory and proliferation capacities of NMCFs stimulated by TGF-β. First of all, the results of cell viability assay showed that low-concentration of β-SITO had no effect on the viability of NMCFs (Figure 4A). Subsequently, we demonstrated that β-SITO suppressed TGF-β-stimulated NMCF proliferation (Figure 4B). What’s more, as presented in Figures 4C,D, β-SITO treatment evidently reduced the mRNA expression of COL-1 and COL-3 compared to the TGF-β-challenged group alone. At the same time, the experimental results suggested that β-SITO reduced the migration of NMCFs induced by TGF-β (Figure 4E). Lastly, immunofluorescence analysis revealed that β-SITO partially attenuated TGF-β-mediated α-SMA enhancement in NMCFs (Figure 4F). Collectively, these findings indicated that β-SITO ameliorated the activation of NMCFs induced by TGF-β *in vitro*.

### 3.5 Transcriptomic insights and molecular docking accounting for the efficacy of β-SITO in the prevention of MI

**FIGURE 5:**
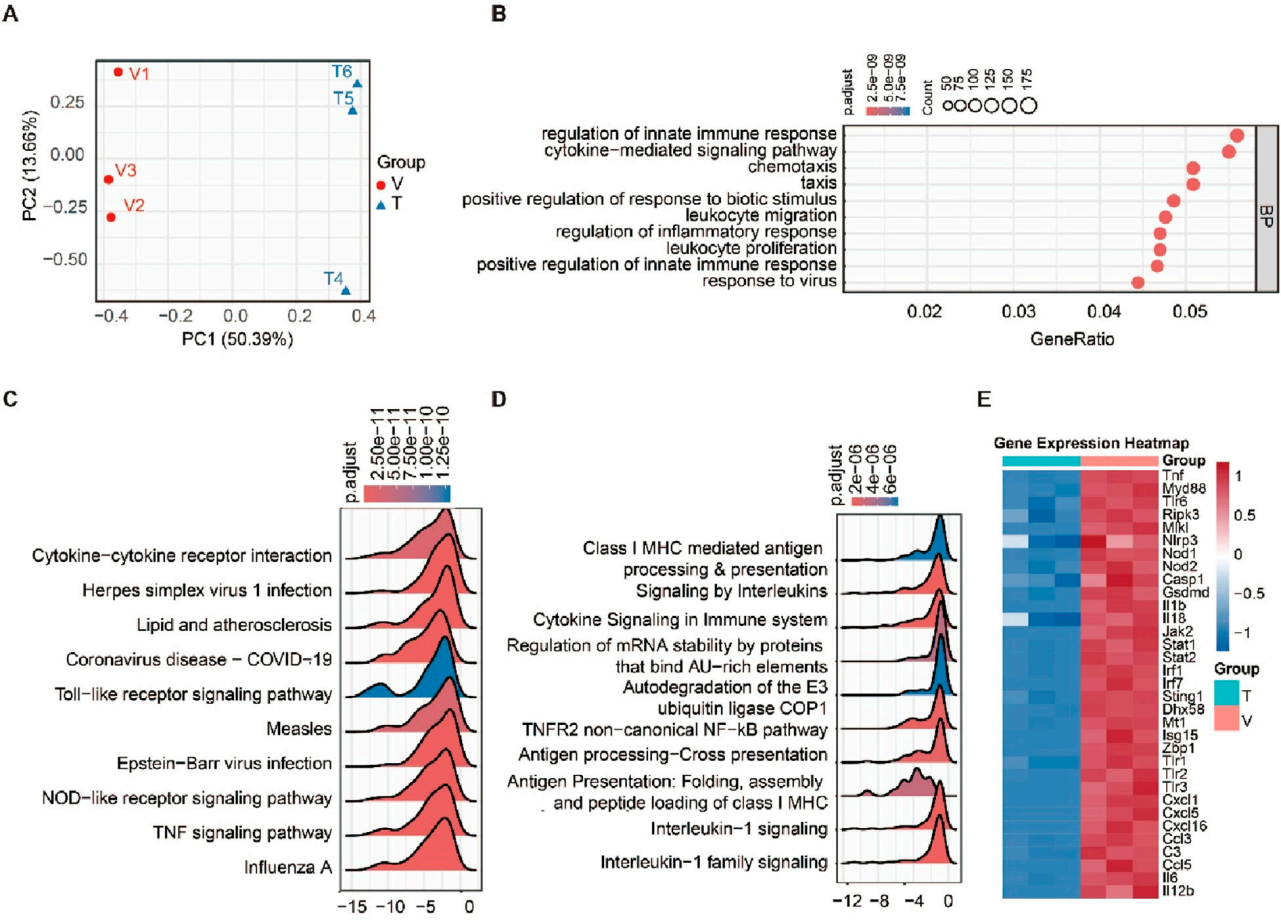
Transcriptomics analysis of β-SITO treatment on MI. **(A)** PCA of transcriptomic profiles in HO + Vehicle (V) and HO + β-SITO group (T). **(B)** Bubble chart of GO enrichment analysis of DEGs between V and T groups. **(C)** The ridge plot of KEGG enrichment of GSEA analysis results across experimental groups. **(D)** Ridge plot displaying the results of GSEA analysis using the Reactome Database. **(E)** The correlation heat map of GSEA analysis results in V and T group. n = 3.

To elucidate β-SITO’s cardioprotective mechanisms, we performed transcriptomic profiling of hypoxic NMCMs treated with either vehicle (V) or β-SITO (T). Initially, Principal component analysis (PCA) revealed significant separation between V and T groups (Figure 5A). Moreover, GO enrichment analysis of DEGs displayed that β-SITO treatment significantly regulated many biological processes associated with immune response and inflammation, such as regulation of innate immune response, leukocyte proliferation and regulation of inflammatory response (Figure 5B). Meanwhile, KEGG pathway enrichment analysis results of GSEA showed that several signaling pathways, such as Toll-like receptor signaling pathway, NOD-like receptor signaling pathway and TNF signaling pathway (Figure 5C). GSEA analysis (Reactome) indicated differential gene enrichment in signaling pathways TNFR2 non-canonical NF-kB pathway, signaling by Interleukins and Cytokine Signaling in Immune system (Figure 5D). Additionally, as described in Figure 5E, the correlation heat map of GSEA analysis revealed that β-SITO treatment effectively reduced the expression of proteins in pathways related to immunity, inflammation, and programmed death, including Nlrp3, IL-1β, IL-6, Cxcl1, Zbp1, Ripk3, Mlkl, Gsdmd, Jak2, Stat1 and Myd88. Of these, as a well-established mediator of programmed cell death, TNF-α triggers RIPK3-dependent necroptosis via TNF-R1. In addition, LPS can activate the ZBP1-RIPK3 axis through TLR4 to induce the same process (Jiao et al., 2020). Considering these findings, we suggested that β-SITO could exerts cardioprotective effects against MI by modulating immune-inflammatory responses and programmed cell death pathways.

**FIGURE 6:**
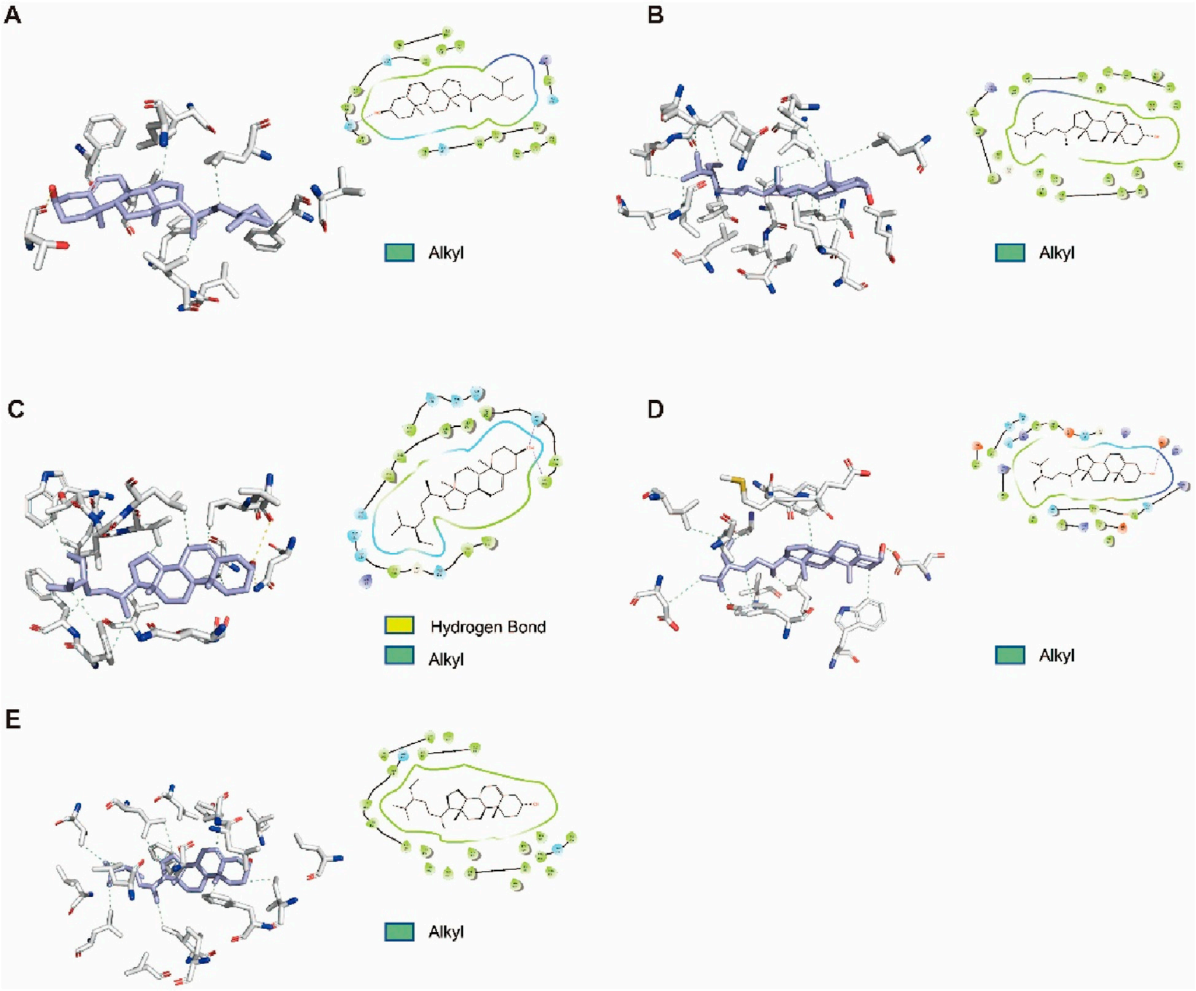
Docking solutions were visualized as both 3D structural models and 2D interaction diagrams. **(A)** P65. **(B)**. SRC3. **(C)**. NLRP3. **(D)**. RIPK3. **(E)**. MLKL.

On basis of the results of RNA-Seq analysis, the NF-κB and necroptosis signaling pathways may be modulated by β-SITO in MI. Subsequently, molecular docking analysis evaluated β-SITO’s binding affinities for specific molecular targets. NF-κB p65, SRC3, NLRP3, RIPK3, and MLKL were selected as target receptors for β-SITO binding prediction, with molecular docking results visualized in Figure 6. The molecular docking scores (kcal/mol) was used to determine the binding strength. Values less than −5 kcal/mol indicate strong binding affinity, and values less than 0 kcal/mol usually indicate spontaneous binding. As shown in Table 1, the binding energies of β-SITO with all target proteins, which ranged from −9.397 to −6.837 kcal/mol, were less than −6.0 kcal/mol, suggesting strong binding affinity. These results demonstrate β-SITO’s high-affinity binding to key targets in NF-κB and necroptosis signaling pathways, while concurrently validating the accuracy of the transcriptome screening.

**TABLE 1:** Docking score.

Protein	Model ID	Molecular docking score (kcal/mol)
p65	AF-Q548Y4-F1	−9.397
SRC3	AF-O09000-F1	−9.294
NLRP3	pdb_00007LFH	−7.467
RIPK3	pdb_00006OKO	−7.465
MLKL	pdb_00004BTF	−6.837

### 3.6 β-SITO represses activation of NF-κB and necroptosis signaling pathways

**FIGURE 7:**
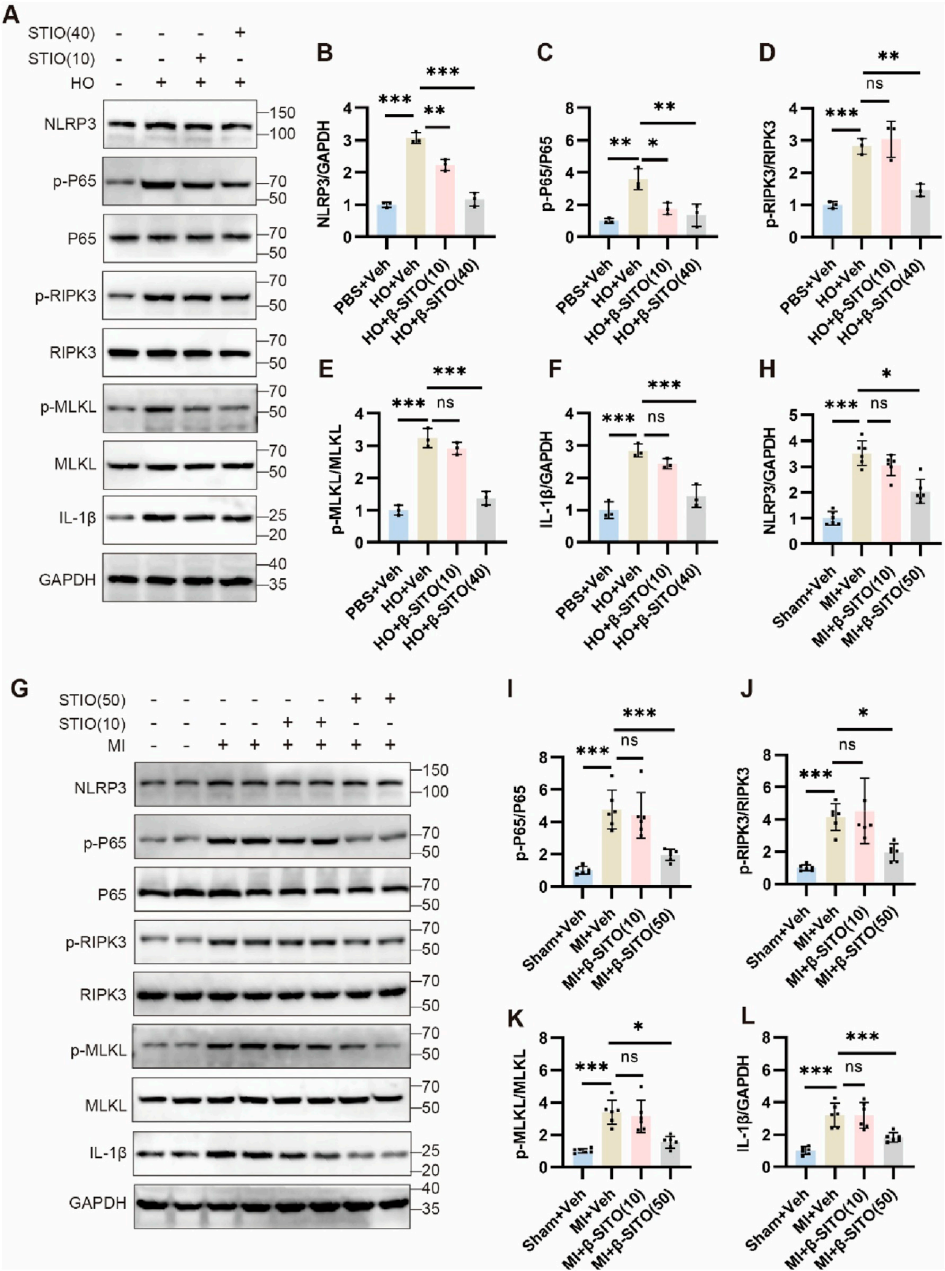
β-SITO represses activation of NF-κB and necroptosis signaling pathways. **(A)** The protein expression levels of NLRP3, p-P65, P65, p-RIPK3, RIPK3, p-MLKL, MLKL and IL-1β were analyzed by immunoblotting in NMCMs. **(B–F)** Protein expression levels were quantified using ImageJ software (n = 3). GAPDH was used as a loading control. **(G)**. The protein expression levels of NLRP3, p-P65, P65, p-RIPK3, RIPK3, p-MLKL, MLKL and IL-1β were analyzed by immunoblotting in mice. **(H–L)** Protein expression levels were quantified using ImageJ software (n = 6). GAPDH was used as a loading control. *P < 0.05, **P < 0.01, ***P < 0.001; ns means no significance.

To validate the key signaling pathways mediating β-SITO’s cardioprotective effects in MI, we analyzed the protein expression of NLRP3 inflammasome components (NLRP3, IL-1β), NF-κB signaling markers (p-P65/P65), and necroptosis executors (p-RIPK3/RIPK3, p-MLKL/MLKL) by Western blotting. As presented in Figures 7A–F, the results suggested that the expression levels of NLRP3, p-P65, p-RIPK3, p-MLKL and IL-1β in HO-induced NMCMs were elevated obviously, but this phenomenon could be reversed by β-SITO administration in a dose-dependent manner. Consistent with *in vitro* findings, β-SITO treatment significantly downregulated myocardial expression of NLRP3, p-P65, p-RIPK3, p-MLKL and IL-1β in MI mice (Figures 7G–L). According to these results, we suggested that β-SITO could ameliorate MI probably via modulating NF-κB and necroptosis signaling pathways.

## 4 Discussion

**FIGURE 8:**
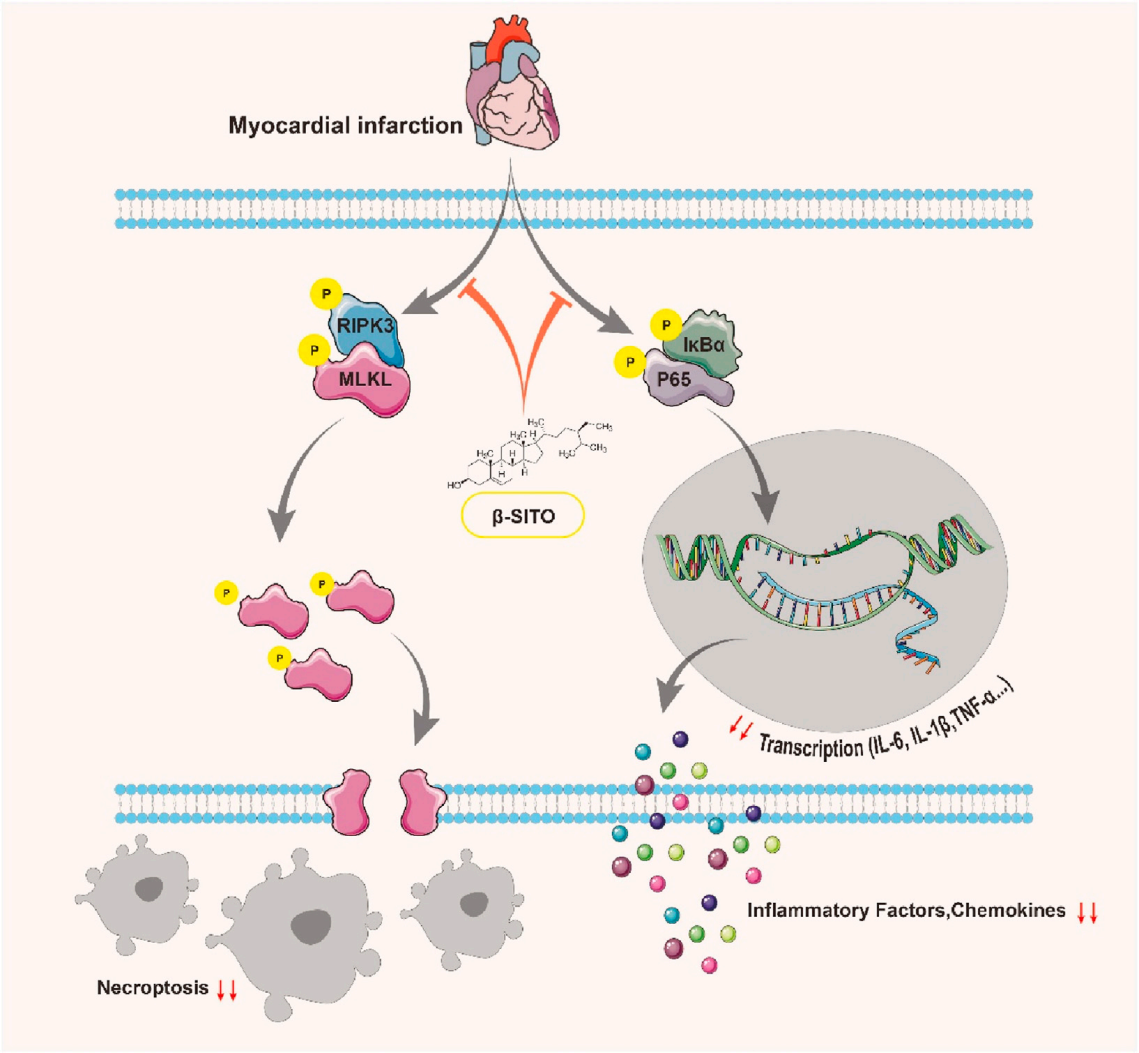
Mechanistic schematic depicting the cardioprotective pathways mediated by β-SITO in MI.

In the present study, we comprehensively investigated the cardioprotective effects and underlying mechanisms of β-SITO in MI using both *in vivo* and *in vitro* models. Our findings demonstrate that β-SITO significantly improves cardiac function, mitigates myocardial fibrosis, reduces cardiomyocyte injury under hypoxic conditions, and inhibits fibroblast activation. Mechanistically, transcriptome sequencing combined with molecular docking and protein validation assays revealed that β-SITO exerts its beneficial effects primarily through the regulation of inflammatory and necroptotic signaling pathways, particularly via modulation of NF-κB, NLRP3 inflammasome, and RIPK3/MLKL-mediated necroptosis (Figure 8).

MI is characterized by ischemic cardiomyocyte death, followed by an inflammatory response and fibrotic remodeling, ultimately leading to progressive cardiac dysfunction and heart failure (Li et al., 2025a). Despite the widespread use of reperfusion therapy and pharmacologic agents such as β-blockers and ACE inhibitors (Chen et al., 2025b), the long-term prognosis for patients with MI remains unsatisfactory, highlighting the need for novel therapeutic approaches targeting the underlying pathophysiology. β-SITO, a plant-derived phytosterol known for its anti-inflammatory and anti-oxidant properties, has emerged as a potential candidate with multifaceted bioactivities (Tang et al., 2024; Jiang et al., 2025). However, its role in cardiovascular disease, particularly in post-MI remodeling, has not been thoroughly elucidated.

Our *in vivo* data provide compelling evidence that β-SITO administration significantly improves left ventricular function post-MI, as evidenced by increased LVEF, LVFS, and cardiac output, alongside reduced infarct size and HW/TL ratios. These functional improvements were accompanied by decreased myocardial expression of hypertrophy markers ANP and BNP, suggesting attenuation of pathological remodeling. Histological analyses further revealed reduced interstitial fibrosis and lower expression of fibrotic markers, including COL-1, COL-3, Galectin-3 and α-SMA, indicating that β-SITO mitigates myocardial fibrosis.

*In vitro*, we demonstrated that β-SITO confers direct cytoprotective effects on NMCMs subjected to hypoxic stress, significantly improving cell viability and reducing cardiomyocytes damage. These results suggest that β-SITO may preserve cardiomyocyte integrity in the ischemic myocardium, thereby preventing the loss of functional myocardial tissue. Additionally, in NMCFs, β-SITO inhibited TGF-β–induced fibroblast proliferation, migration, and myofibroblast differentiation. These findings demonstrate a dual mechanism by which β-SITO acts—protecting cardiomyocytes and preventing excessive fibroblast activation, thereby limiting both cell loss and fibrotic scar expansion.

To investigate the cardioprotective mechanisms of β-SITO, we conducted RNA-seq analysis of HO-induced NMCMs treated with either vehicle or β-SITO. Functional enrichment of DEGs indicated significant involvement of inflammation- and cell death-related pathways, including NF-κB signaling, cytokine-cytokine receptor interaction, and necroptosis. Notably, KEGG and GO analyses highlighted the involvement of genes such as NLRP3, RIPK3, MLKL, and IL-1β. Moreover, the interaction between β-SITO and the identified targets was further supported by molecular docking analysis, which showed favorable binding affinities between β-SITO and NLRP3, RIPK3, MLKL, and P65. These *in silico* findings provide a mechanistic rationale for the observed effects and suggest that β-SITO may function as a direct modulator of key inflammatory and cell death signaling pathways.

The NLRP3 inflammasome plays a critical role in sterile inflammation following MI by promoting the maturation and release of pro-inflammatory cytokines, notably IL-1β, thereby exacerbating cardiomyocyte injury and fibrosis (Cui et al., 2025). In this study, β-SITO treatment significantly suppressed NLRP3 and IL-1β expression in both *in vivo* and *in vitro* models, suggesting that inhibition of inflammasome activation is a central mechanism of its cardioprotective effect. Additionally, β-SITO reduced the phosphorylation of NF-κB p65, key mediators of canonical NF-κB activation, further confirming its anti-inflammatory potential. These findings are consistent with previous reports of β-SITO’s anti-inflammatory activity in other disease contexts, such as metabolic and neuroinflammatory disorders (Li X. et al., 2025; Dolrahman and Thong-Asa, 2024).

Necroptosis, a regulated form of necrotic cell death mediated by the RIPK3-MLKL axis, has recently emerged as a major contributor to cardiomyocyte death during ischemia-reperfusion injury, MI and heart failure (Qin et al., 2024). In our study, β-SITO markedly reduced p-RIPK3 and p-MLKL expression, indicating inhibition of necroptotic signaling. This finding is particularly significant given that necroptosis not only contributes to myocardial cell loss but also promotes inflammation through the release of damage-associated molecular patterns (DAMPs). By suppressing this pathway, β-SITO likely exerts both cytoprotective and anti-inflammatory effects, creating a more favorable environment for myocardial healing.

Collectively, our study provides robust evidence that β-SITO mitigates myocardial injury and fibrosis following MI through a multifaceted mechanism involving suppression of inflammation and necroptosis. These effects are mediated, at least in part, by direct inhibition of the NLRP3 inflammasome and RIPK3/MLKL pathway, as well as modulation of NF-κB signaling. Importantly, these molecular effects translate into significant improvements in cardiac structure and function, highlighting the translational potential of β-SITO as a therapeutic agent.

## 5 Limitation

Despite these promising findings, several limitations warrant consideration. First, while we demonstrated the efficacy of β-SITO in a murine model of MI, further studies in large animal models are needed to validate its therapeutic potential and determine optimal dosing strategies. Second, although molecular docking provides insight into potential binding interactions, confirmatory studies such as surface plasmon resonance or co-immunoprecipitation are needed to validate direct target engagement. Lastly, the bioavailability and pharmacokinetics of β-SITO in the context of cardiac injury remain to be fully characterized.

## 6 Conclusion

Our findings demonstrate that β-SITO is a promising cardioprotective agent capable of mitigating myocardial injury and remodeling through the suppression of inflammation and necroptosis. These results pave the way for future studies aimed at translating β-SITO into a viable therapeutic strategy for patients with MI and potentially other forms of cardiovascular disease.

## Data Availability

The original contributions presented in the study are publicly available. This data can be found here: NCBI GEO repository, accession number GSE314346.

## References

[B1] Abramson J. Adler J. Dunger J. Evans R. Green T. Pritzel A. (2024). Accurate structure prediction of biomolecular interactions with AlphaFold 3. Nature 630 (8016), 493–500. 10.1038/s41586-024-07487-w 38718835 PMC11168924

[B2] Adhimoolam K. Sureshbabu A. Smirnova E. Muthuramalingam P. Do T. C. Senthil K. (2024). beta-Sitosterol-Dietary sources and role in cancer and diabetes management. Food Sci. Nutr. 12 (11), 8870–8886. 10.1002/fsn3.4380 39619995 PMC11606809

[B3] Alghibiwi H. K. Alhusiani A. M. Sarawi W. S. Fadda L. Alomar H. A. Alsaab J. S. (2025). Coenzyme Q10 and its liposomal form prevent copper cardiotoxicity by attenuating oxidative stress, TLR-4/NF-kappaB signaling and necroptosis in rats. Cell Mol. Biol. (Noisy-le-grand). 71 (1), 118–124. 10.14715/cmb/2025.70.1.13 39910933

[B4] Chai R. Ye Z. Xue W. Shi S. Wei Y. Hu Y. (2023). Tanshinone IIA inhibits cardiomyocyte pyroptosis through TLR4/NF-kappaB p65 pathway after acute myocardial infarction. Front. Cell Dev. Biol. 11, 1252942. 10.3389/fcell.2023.1252942 37766966 PMC10520722

[B5] Chapman A. R. Taggart C. Boeddinghaus J. Mills N. L. Fox K. (2025). Type 2 myocardial infarction: challenges in diagnosis and treatment. Eur. Heart J. 46 (6), 504–517. 10.1093/eurheartj/ehae803 39658094 PMC11804249

[B6] Chen Y. Yang Y. Wang N. Liu R. Wu Q. Pei H. (2024). beta-Sitosterol suppresses hepatocellular carcinoma growth and metastasis via FOXM1-regulated Wnt/beta-catenin pathway. J. Cell. Mol. Med. 28 (3), e18072. 10.1111/jcmm.18072 38063438 PMC10844700

[B7] Chen L. Zeng L. Pan S. Zu L. Pan H. Fan L. (2025). beta-sitosterol in yijing hugui decoction prevents cyclophosphamide-induced premature ovarian insufficiency via the AKT1/Nrf2 pathway. Cytotechnology 77 (2), 76. 10.1007/s10616-025-00740-8 40078376 PMC11893954

[B8] Chen S. Zeng X. Wu M. Zhu J. Wu Y. (2025). Sodium alginate hydrogel infusion of bone marrow mesenchymal stem cell-derived extracellular vesicles and p38alpha antagonistic peptides in myocardial infarction fibrosis mitigation. J. Am. Heart Assoc. 14 (8), e036887. 10.1161/JAHA.124.036887 40178108 PMC12184616

[B9] Cui L. G. Wang S. H. Komal S. Yin J. J. Zhai M. M. Zhou Y. J. (2025). ALKBH5 promotes cardiac fibroblasts pyroptosis after myocardial infarction through Notch1/NLRP3 pathway. Cell. Signal. 127, 111574. 10.1016/j.cellsig.2024.111574 39710090

[B10] Dolrahman N. Thong-Asa W. (2024). Beta-sitosterol mitigates cognitive deficit and hippocampal neurodegeneration in mice with trimethyltin-induced toxicity. Exp. Anim. 73 (4), 433–445. 10.1538/expanim.24-0021 38945945 PMC11534485

[B11] Galluzzi L. Vitale I. Aaronson S. A. Abrams J. M. Adam D. Agostinis P. (2018). Molecular mechanisms of cell death: recommendations of the nomenclature committee on cell Death 2018. Cell Death Differ. 25 (3), 486–541. 10.1038/s41418-017-0012-4 29362479 PMC5864239

[B12] Gao X. Ni C. Song Y. Xie X. Zhang S. Chen Y. (2025). Dan-shen Yin attenuates myocardial fibrosis after myocardial infarction in rats: molecular mechanism insights by integrated transcriptomics and network pharmacology analysis and experimental validation. J. Ethnopharmacol. 338 (Pt 2), 119070. 10.1016/j.jep.2024.119070 39522849

[B13] Gupta R. Sharma A. K. Dobhal M. P. Sharma M. C. Gupta R. S. (2011). Antidiabetic and antioxidant potential of beta-sitosterol in streptozotocin-induced experimental hyperglycemia. J. Diabetes 3 (1), 29–37. 10.1111/j.1753-0407.2010.00107.x 21143769

[B14] Jacobson M. P. Friesner R. A. Xiang Z. Honig B. (2002). On the role of the crystal environment in determining protein side-chain conformations. J. Mol. Biol. 320 (3), 597–608. 10.1016/s0022-2836(02)00470-9 12096912

[B15] Jacobson M. P. Pincus D. L. Rapp C. S. Day T. J. Honig B. Shaw D. E. (2004). A hierarchical approach to all-atom protein loop prediction. Proteins 55 (2), 351–367. 10.1002/prot.10613 15048827

[B16] Jaiswal S. Anjum M. M. Arya D. K. Thakur S. Pandey P. Deepak P. (2024). Surface entrenched beta-sitosterol niosomes for enhanced cardioprotective activity against isoproterenol induced cardiotoxicity in rats. Int. J. Pharm. 653, 123872. 10.1016/j.ijpharm.2024.123872 38336178

[B17] Jayaraman S. Prasad M. Natarajan S. R. Krishnamoorthy R. Alshuniaber M. A. Gatasheh M. K. (2025). Molecular mechanisms underlying the effects of beta-sitosterol on TGF-beta1/Nrf2/SIRT1/p53-mediated signaling in the kidney of a high-fat diet and sucrose-induced type-2 diabetic rat. Chem. Biol. Interact. 411, 111443. 10.1016/j.cbi.2025.111443 39986364

[B18] Jiang S. Gao K. Zhang F. Wang Y. He X. Yang J. (2024). beta-sitosterol alleviates atherosclerosis by regulating catalase. Heliyon 10 (15), e35639. 10.1016/j.heliyon.2024.e35639 39165938 PMC11334795

[B19] Jiang L. Sun X. Wan Y. Qin Q. Xu M. Ma J. (2025). Transcriptome reveals the promoting effect of beta-sitosterol on the differentiation of bovine preadipocytes. J. Agric. Food Chem. 73 (6), 3400–3412. 10.1021/acs.jafc.4c10452 39874185

[B20] Jiao H. Wachsmuth L. Kumari S. Schwarzer R. Lin J. Eren R. O. (2020). Z-nucleic-acid sensing triggers ZBP1-dependent necroptosis and inflammation. Nature 580 (7803), 391–395. 10.1038/s41586-020-2129-8 32296175 PMC7279955

[B21] Kim J. H. Duan S. Baik M. Y. Eom S. H. (2025). Thermal stability of stigmasterol and beta-sitosterol glucosides in fresh-cut bitter melon fruit. Food Chem. 468, 142414. 10.1016/j.foodchem.2024.142414 39671920

[B22] Li J. Meng Z. Y. Wen H. Lu C. H. Qin Y. Xie Y. M. (2024). beta-sitosterol alleviates pulmonary arterial hypertension by altering smooth muscle cell phenotype and DNA damage/cGAS/STING signaling. Phytomedicine 135, 156030. 10.1016/j.phymed.2024.156030 39265206

[B23] Li H. Gong Y. Wang Y. Sang W. Wang C. Zhang Y. (2025). beta-Sitosterol modulates osteogenic and adipogenic balance in BMSCs to suppress osteoporosis via regulating mTOR-IMP1-Adipoq axis. Phytomedicine 138, 156400. 10.1016/j.phymed.2025.156400 39848018

[B24] Li X. Zhu X. Jiang S. Yang W. Zhang F. Guo X. (2025b). Atractylenolide-III restrains cardiac fibrosis after myocardial infarction via suppression of the RhoA/ROCK1 and ERK1/2 pathway. Int. Immunopharmacol. 145, 113825. 10.1016/j.intimp.2024.113825 39667049

[B25] Liu Y. Li Z. Li W. Chen X. Yang L. Lu S. (2024). Discovery of beta-sitosterol's effects on molecular changes in rat diabetic wounds and its impact on angiogenesis and macrophages. Int. Immunopharmacol. 126, 111283. 10.1016/j.intimp.2023.111283 38035407

[B26] Lu C. Y. Lu P. C. Chen P. C. (2019). Utilization trends in traditional Chinese medicine for acute myocardial infarction. J. Ethnopharmacol. 241, 112010. 10.1016/j.jep.2019.112010 31175928

[B27] Lu C. Wu C. Ghoreishi D. Chen W. Wang L. Damm W. (2021). OPLS4: improving force field accuracy on challenging regimes of chemical space. J. Chem. Theory Comput. 17 (7), 4291–4300. 10.1021/acs.jctc.1c00302 34096718

[B28] Luedde M. Lutz M. Carter N. Sosna J. Jacoby C. Vucur M. (2014). RIP3, a kinase promoting necroptotic cell death, mediates adverse remodelling after myocardial infarction. Cardiovasc. Res. 103 (2), 206–216. 10.1093/cvr/cvu146 24920296

[B29] Ma Z. Li M. Guo R. Tian Y. Zheng Y. Huang B. (2025). Treating myocardial infarction via a nano-ultrasonic contrast agent-mediated high-efficiency drug delivery system targeting macrophages. Sci. Adv. 11 (1), eadp7126. 10.1126/sciadv.adp7126 39752485 PMC11698097

[B30] Maslov L. N. Popov S. V. Naryzhnaya N. V. Mukhomedzyanov A. V. Kurbatov B. K. Derkachev I. A. (2022). The regulation of necroptosis and perspectives for the development of new drugs preventing ischemic/reperfusion of cardiac injury. Apoptosis 27 (9-10), 697–719. 10.1007/s10495-022-01760-x 35986803

[B31] Mekarunothai A. Bacher M. Buathong R. Intarasam S. Tayana N. Kongkiatpaiboon S. (2024). beta-sitosterol isolated from the leaves of Trema orientalis (Cannabaceae) promotes viability and proliferation of BF-2 cells. PeerJ 12, e16774. 10.7717/peerj.16774 38282858 PMC10812590

[B32] Napolitano G. Ballabio A. (2016). TFEB at a glance. J. Cell Sci. 129 (13), 2475–2481. 10.1242/jcs.146365 27252382 PMC4958300

[B33] Qin D. Modanwal R. Kitsis R. N. (2024). Extracellular role for the intracellular cell death mediator RIPK3 in myocardial infarction. Circulation 150 (22), 1812–1814. 10.1161/CIRCULATIONAHA.124.072172 39585932 PMC11594480

[B34] Roth G. A. Mensah G. A. Johnson C. O. Addolorato G. Ammirati E. Baddour L. M. (2020). Global burden of cardiovascular diseases and risk factors, 1990-2019: update from the GBD 2019 study. J. Am. Coll. Cardiol. 76 (25), 2982–3021. 10.1016/j.jacc.2020.11.010 33309175 PMC7755038

[B35] Shan T. Li X. Xie W. Wang S. Gao Y. Zheng Y. (2024). Rap1GAP exacerbates myocardial infarction by regulating the AMPK/SIRT1/NF-kappaB signaling pathway. Cell. Signal. 117, 111080. 10.1016/j.cellsig.2024.111080 38320624

[B36] Sun X. Chen J. Shang J. Liu H. Li X. Lou Y. (2024). Traditional chinese medicine injections with activating blood circulation, equivalent effect of anticoagulation or antiplatelet, for acute myocardial infarction: a systematic review and meta-analysis of randomized clinical trials. Complement. Ther. Med. 82, 103039. 10.1016/j.ctim.2024.103039 38616000

[B37] Tang X. Yan T. Wang S. Liu Q. Yang Q. Zhang Y. (2024). Treatment with beta-sitosterol ameliorates the effects of cerebral ischemia/reperfusion injury by suppressing cholesterol overload, endoplasmic reticulum stress, and apoptosis. Neural Regen. Res. 19 (3), 642–649. 10.4103/1673-5374.380904 37721296 PMC10581587

[B38] Wang H. Liu J. Zhang Z. Peng J. Wang Z. Yang L. (2024). beta-Sitosterol targets ASS1 for Nrf2 ubiquitin-dependent degradation, inducing ROS-mediated apoptosis via the PTEN/PI3K/AKT signaling pathway in ovarian cancer. Free Radic. Biol. Med. 214, 137–157. 10.1016/j.freeradbiomed.2024.02.004 38364944

[B39] Wang L. H. Sun Y. H. Liu H. Yang X. Wen Z. Tian X. F. (2024). beta-Sitosterol attenuates anlotinib resistance in non-small cell lung cancer cells by inhibiting miR-181a-3p/SHQ1 signaling. Chem. Biol. Drug Des. 103 (3), e14493. 10.1111/cbdd.14493 38439529

[B40] Wang C. Xu W. Jiang S. Wu Y. Shu J. Gao X. (2025). beta-Hydroxybutyrate facilitates postinfarction cardiac repair via targeting PHD2. Circ. Res. 136 (7), 704–718. 10.1161/CIRCRESAHA.124.325179 39957619

[B41] Wang J. Zhou M. Zhou Q. Sun G. Zhang Y. Tao F. (2025). Beta-sitosterol regulates PTGS1 to inhibit gastric cancer cell proliferation and angiogenesis. Prostagl. Other Lipid Mediat 177, 106964. 10.1016/j.prostaglandins.2025.106964 39863019

[B42] Wang Q. Yang Y. Yuan X. Luo F. Ling Y. Chen C. (2025). Jinwu jiangu capsule alleviates rheumatoid arthritis symptoms by regulating the ADCY10 and cAMP/RANKL pathways. J. Ethnopharmacol. 338 (Pt 3), 119099. 10.1016/j.jep.2024.119099 39542189

[B43] Wang W. Li L. Li X. Chen J. Wang R. Yang Q. (2025). beta-Sitosterol protects against lithocholic acid-induced hepatotoxicity and cholestasis via farnesoid X receptor-mediated regulation of transporters and enzymes *in vitro* and *in vivo* . Toxicol. Appl. Pharmacol. 498, 117308. 10.1016/j.taap.2025.117308 40120651

[B44] Wang Y. Zheng Y. Liang X. Chang Y. Liu Y. Cheng X. (2025). alpha-Lipoic acid alleviate myocardial infarction by suppressing age-independent macrophage senescence. Sci. Rep. 15 (1), 11996. 10.1038/s41598-025-92328-7 40199978 PMC11978910

[B45] Wei W. Xu G. Gao J. Wang G. Wang Y. Li C. (2025). Sacubitril/Valsartan partially alleviates myocardial infarction injury by activating the FGF21 signaling pathway via PPARs. Cardiovasc. Diabetol. 24 (1), 89. 10.1186/s12933-025-02627-6 39987117 PMC11847388

[B46] Wu H. Lan Q. He Y. X. Xue J. Y. Liu H. Zou Y. (2025). Programmed cardiomyocyte death in myocardial infarction. Apoptosis 30 (3-4), 597–615. 10.1007/s10495-025-02075-3 39833636

[B47] Wu Y. Ge H. Zhao H. Zou K. Wang P. Wang Y. (2025). The active ingredient beta-sitosterol in the anti-inflammatory agents alleviates perianal inflammation in rats by inhibiting the expression of Srebf2, activating the PPAR signaling pathway, and altering the composition of gut microbiota. Int. Immunopharmacol. 152, 114470. 10.1016/j.intimp.2025.114470 40086059

[B48] Yan D. Zhan S. Guo C. Han J. Zhan L. Zhou Q. (2025). The role of myocardial regeneration, cardiomyocyte apoptosis in acute myocardial infarction: a review of current research trends and challenges. J. Cardiol. 85 (4), 283–292. 10.1016/j.jjcc.2024.09.012 39393490

[B49] Yang C. Zhu C. Li Y. Li Z. Zhang Z. Xu J. (2022). Injectable selenium-containing polymeric hydrogel formulation for effective treatment of myocardial infarction. Front. Bioeng. Biotechnol. 10, 912562. 10.3389/fbioe.2022.912562 36032710 PMC9403312

[B50] Yang M. Q. Chen C. Mao Y. F. Li Y. Zhong X. Yu Y. D. (2022). Application of network pharmacology and molecular docking approach to explore active compounds and potential pharmacological mechanisms of Aconiti Lateralis Radix praeparata and lepidii semen descurainiae semen for treatment of heart failure. Med. Baltim. 101 (33), e30102. 10.1097/MD.0000000000030102 35984130 PMC9387970

[B51] Yang Y. Zhang Y. Yang J. Zhang M. Tian T. Jiang Y. (2023). Interdependent nuclear Co-Trafficking of ASPP1 and p53 aggravates cardiac ischemia/reperfusion injury. Circ. Res. 132 (2), 208–222. 10.1161/CIRCRESAHA.122.321153 36656967 PMC9855749

[B52] Yang W. Tian Y. Yang M. Mauck J. Loor J. J. Jia B. (2024). beta-sitosterol alleviates high fatty acid-induced lipid accumulation in calf hepatocytes by regulating cholesterol metabolism. J. Steroid Biochem. Mol. Biol. 243, 106543. 10.1016/j.jsbmb.2024.106543 38740074

[B53] Yang S. Zhang Y. Zheng C. (2025). beta-Sitosterol mitigates apoptosis, oxidative stress and inflammatory response by inactivating TLR4/NF-small ka, CyrillicB pathway in cell models of diabetic nephropathy. Cell biochem. Biophys. 83 (1), 1249–1262. 10.1007/s12013-024-01559-4 39424766

[B54] Zhang D. Y. Wang B. J. Ma M. Yu K. Zhang Q. Zhang X. W. (2019). MicroRNA-325-3p protects the heart after myocardial infarction by inhibiting RIPK3 and programmed necrosis in mice. BMC Mol. Biol. 20 (1), 17. 10.1186/s12867-019-0133-z 31248365 PMC6598367

[B55] Zhang Y. Zhang Y. Zang J. Li Y. Wu X. (2023). Pharmaceutical therapies for necroptosis in myocardial ischemia-reperfusion injury. J. Cardiovasc Dev. Dis. 10 (7), 303. 10.3390/jcdd10070303 37504559 PMC10380972

[B56] Zhang P. Liu N. Xue M. Zhang M. Xiao Z. Xu C. (2024). beta-Sitosterol reduces the content of triglyceride and cholesterol in a high-fat diet-induced non-alcoholic fatty liver disease zebrafish (Danio rerio) model. Anim. (Basel) 14 (9), 1289. 10.3390/ani14091289 38731293 PMC11083524

[B57] Zhang G. Sun X. Zhang D. Zhang X. Yu K. (2025). SerpinA3 promotes myocardial infarction in rat and cell-based models. Mol. Biotechnol. 67 (1), 92–103. 10.1007/s12033-023-00982-x 38006519

[B58] Zhang X. Yang L. Feng K. Zhang H. Chen Y. Li W. (2025). Shuxuening injection improves myocardial injury after myocardial infarction by regulating macrophage polarization via the TLR4/NF-kappaB and PI3K/Akt signaling pathways. Phytomedicine 138, 156418. 10.1016/j.phymed.2025.156418 39879705

[B59] Zhao D. Liu J. Wang M. Zhang X. Zhou M. (2019). Epidemiology of cardiovascular disease in China: current features and implications. Nat. Rev. Cardiol. 16 (4), 203–212. 10.1038/s41569-018-0119-4 30467329

[B60] Zhu L. Liu Y. Wang K. Wang N. (2025). Regulated cell death in acute myocardial infarction: molecular mechanisms and therapeutic implications. Ageing Res. Rev. 104, 102629. 10.1016/j.arr.2024.102629 39644925

[B61] Zhuang L. Zong X. Yang Q. Fan Q. Tao R. (2023). Interleukin-34-NF-kappaB signaling aggravates myocardial ischemic/reperfusion injury by facilitating macrophage recruitment and polarization. EBioMedicine 95, 104744. 10.1016/j.ebiom.2023.104744 37556943 PMC10433018

