# Supplementary material for: β-sitosterol ameliorates myocardial infarction injury via modulating the NF-κB and necroptosis signaling pathways
Source: Front Pharmacol. 2026 Jan 6;16:1719074. doi: 10.3389/fphar.2025.1719074 (PMC12816229; doi:10.3389/fphar.2025.1719074)
Supplement: Supplementary file 1 [file Table1.docx]

**Supplementary Table S1.** **The list of primer**

| Gene | Sequence |
| --- | --- |
| Mouse ANP Forward | 5’-GGAGCCTACGAAGATCCAGC-3’ |
| Reverse | 5’-TCCAATCCTGTCAATCCTACCC-3’ |
| Mouse BNP Forward | 5’-CTTCGGTCTCAAGGCAGCAC-3’ |
| Reverse | 5’-GCCCAAACGACTGACGGATC-3’ |
| Mouse Galectin-3 Forward | 5’-CAGGAAAATGGCAGACAGCTT-3’ |
| Reverse | 5’- CCCATGCACCCGGATATC-3’ |
| Mouse Collagen I Forward | 5’-ATGGATTCCCGTTCGAGTAC-3’ |
| Reverse | 5’-TCAGCTGGATAGCGACATCG-3’ |
| Mouse Collagen III Forward | 5’-CGTAGATGAATTGGGATGCA-3’ |
| Reverse | 5’-ACATGGTTCTGGCTTCCAG-3’ |
| Mouse a-SMA Forward | 5’-TGCTGACAGAGGCACCACTGAA-3’ |
| Reverse | 5’- CAGTTGTACGTCCAGAGGCATAG-3’ |
| Mouse GAPDH Forward | 5’-TGGCCTTCCGTGTTCCTAC-3’ |
| Reverse | 5’ -GAGTTGCTGTTGAAGTCGCA-3’ |
