# Supplementary figures and images for: β-sitosterol ameliorates myocardial infarction injury via modulating the NF-κB and necroptosis signaling pathways
Source: Front Pharmacol. 2026 Jan 6;16:1719074. doi: 10.3389/fphar.2025.1719074 (PMC12816229; doi:10.3389/fphar.2025.1719074)

Fig.8A

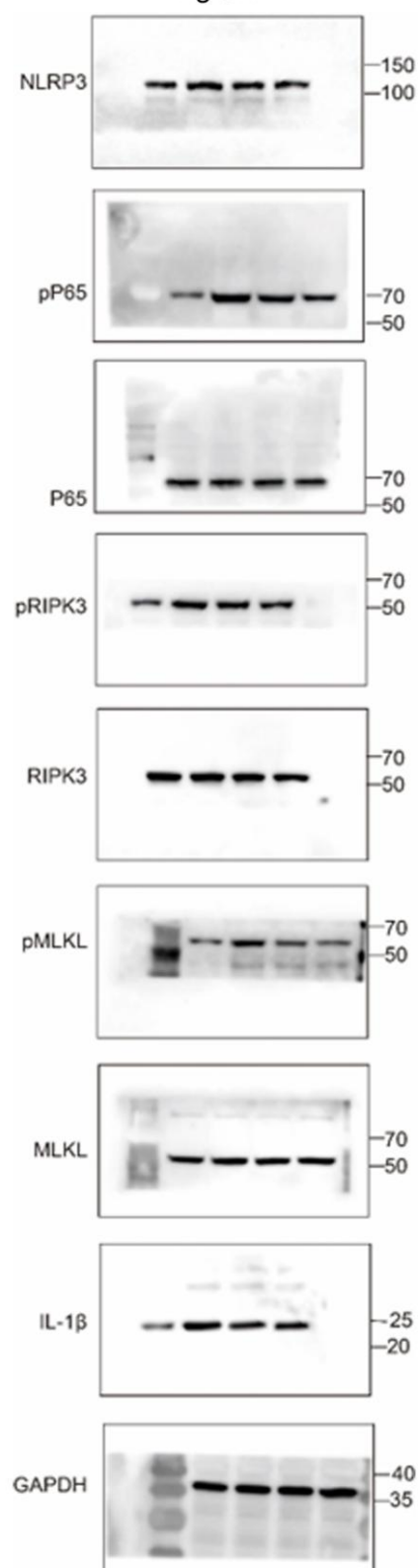

Fig.8G

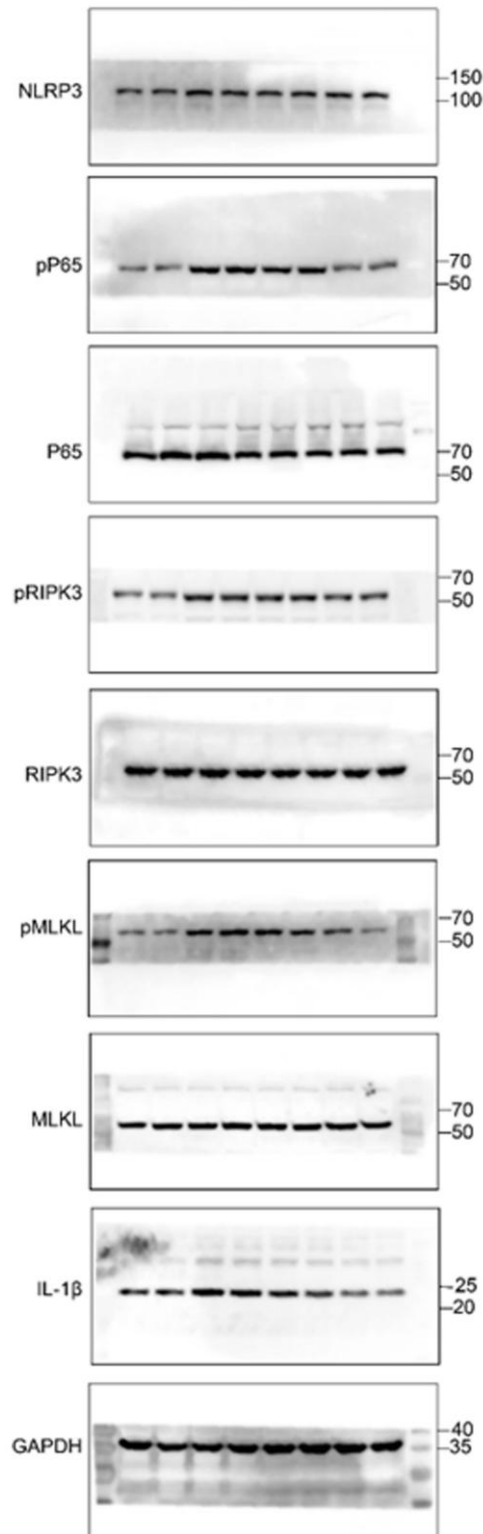

Supplement: Supplementary file 2 [file DataSheet1.pdf]
